# CD28 Autonomous Signaling Orchestrates IL-22 Expression and IL-22-Regulated Epithelial Barrier Functions in Human T Lymphocytes

**DOI:** 10.3389/fimmu.2020.590964

**Published:** 2020-10-14

**Authors:** Martina Kunkl, Carola Amormino, Simone Frascolla, Manolo Sambucci, Marco De Bardi, Silvana Caristi, Stefano Arcieri, Luca Battistini, Loretta Tuosto

**Affiliations:** ^1^ Department of Biology and Biotechnology Charles Darwin, Sapienza University, Rome, Italy; ^2^ Laboratory affiliated to Istituto Pasteur Italia-Fondazione Cenci Bolognetti, Sapienza University, Rome, Italy; ^3^ Neuroimmunology Unit, IRCCS Santa Lucia Foundation, Rome, Italy; ^4^ Department of Surgical Sciences, Sapienza University of Rome, Rome, Italy

**Keywords:** CD28, IL-22, epithelial cell, T cell, inflammation

## Abstract

IL-22 is a member of the IL-10 cytokine family involved in host protection against extracellular pathogens, by promoting epithelial cell regeneration and barrier functions. Dysregulation of IL-22 production has also frequently been observed in acute respiratory distress syndrome (ARDS) and several chronic inflammatory and autoimmune diseases. We have previously described that human CD28, a crucial co-stimulatory receptor necessary for full T cell activation, is also able to act as a TCR independent signaling receptor and to induce the expression of IL-17A and inflammatory cytokines related to Th17 cells, which together with Th22 cells represent the main cellular source of IL-22. Here we characterized the role of CD28 autonomous signaling in regulating IL-22 expression in human CD4^+^ T cells. We show that CD28 stimulation in the absence of TCR strongly up-regulates IL-22 gene expression and secretion. As recently observed for IL-17A, we also found that CD28-mediated regulation of IL-22 transcription requires the cooperative activities of both IL-6-activated STAT3 and RelA/NF-κB transcription factors. CD28-mediated IL-22 production also promotes the barrier functions of epithelial cells by inducing mucin and metalloproteases expression. Finally, by using specific inhibitory drugs, we also identified CD28-associated class 1A phosphatidylinositol 3-kinase (PI3K) as a pivotal mediator of CD28-mediated IL-22 expression and IL-22–dependent epithelial cell barrier functions.

## Introduction

IL-22 has been discovered in 2000 as a member of the IL-10 family that affects the functions of several non-hematopoietic cells, such as epithelial cells, keratinocytes, and hepatocytes (1). IL-22 is mainly produced by Th17 cells, a subset of CD4^+^ T helper (Th) cells that regulate efficient immune defense against extracellular pathogens (2) and by a unique CD4^+^ Th cell subset, named Th22 (3–5). Other cell types have been described to produce IL-22 such as natural killer (NK) cells, ILC3 subset of innate lymphoid cells and at lower extent macrophages (6–8). IL-22 elicits its main effect on epithelial cells by stimulating their regeneration and proliferation, and by promoting epithelial barrier functions essential for the host defense against extracellular microbes at mucosal surfaces (9–14). However, high levels of IL-22 and IL-17 were detected in plasma of patients with sepsis-induced acute respiratory distress syndrome (ARDS) and were correlated with poor prognosis (15). More recently, a novel severe acute respiratory syndrome-related coronavirus 2 (SARS-Cov2) has been identified as the etiological agent of COVID-19 (16), a respiratory pandemic disease that, in several patients, causes ARDS associated with high levels of pro-inflammatory cytokines (17) including Th17 cytokines such as IL-22, IL-17, and IL-6 (18, 19). Moreover, an excessive production of IL-22 together with other cytokines of the Th17 signature has also been involved in maintaining and amplifying the chronic state of several inflammatory and autoimmune diseases (20–26). Therefore, the identification of stimulatory molecules and associated signaling pathways regulating and/or amplifying IL-22 production may provide new insight into immune defense against pathogens and novel therapeutic targets for inflammatory and autoimmune diseases.

CD28 is one of the most important costimulatory receptor required for full T cell activation and differentiation (27). CD28 interacts with B7.1/CD80 or B7.2/CD86 on the surface of professional antigen presenting cells (APCs) and lowers the threshold of the T cell receptor (TCR), thus enhancing the early signaling events necessary for optimal cytokine production, cell cycle progression and survival (28, 29). Human CD28 is also able to deliver TCR-independent signals, which leads to the production of pro-inflammatory cytokines and chemokines (30, 31) in CD4^+^ T cells. We also evidenced that CD28 autonomous stimulation of peripheral blood CD4^+^ T cells from either healthy donors (HD) or type 1 diabetes (T1D) or multiple sclerosis (MS) patients promotes the production of inflammatory cytokines and chemokines related to the Th17 cell phenotype (32–34), including IL-22 (35).

In this work, we elucidated the role of CD28 autonomous stimulation in the regulation of IL-22 expression and IL-22–mediated effector functions. We found that IL-22 gene expression and secretion was strongly up-regulated by CD28 in human CD4^+^ T cells. Similar to IL-17A, CD28-induced IL-22 gene expression was regulated by both RelA/NF-κB and IL-6–regulated STAT3 transcription factors. Co-culture experiments of CD28-activated T cells with CACO-2 epithelial cell lines evidenced a critical role of IL-22 in regulating the expression of mucins and metalloproteases. Finally, we also evidence a pivotal role of CD28-associated class 1A phosphatidylinositol 3-kinase (PI3K) in regulating both IL-22 expression and IL-22–dependent epithelial barrier functions.

## Materials and Methods

### Cells, Abs, and Reagents

Human primary CD4^+^ T cells were enriched from PBMC by negative selection using an EasySepTM isolation kit (#17952, STEMCELL Technology) and cultured in RPMI 1640 supplemented with 5% human serum (Euroclone, UK), L-glutamine, penicillin and streptomycin. The purity of the sorted population was 95% to 99%, as evidenced by staining with anti-CD3 plus anti-CD4 Abs. Human naïve CD4^+^CD45RA^+^ and effector/memory CD4^+^CD45RO^+^ T cells were sorted using a high speed cell sorter (Moflo Astrios EQ, Beckman Coulter). Purity of sorted cells was consistently > 98%, and was acquired on a cytometer (CytoFLEX, Beckman Coulter). PBMCs were derived from buffy coats or anonymous healthy blood (HD) donors provided by the Policlinico Umberto I (Sapienza University of Rome, Italy). Written informed consent was obtained from blood donors and both the informed consent form and procedure was approved by the Ethics Committee of Policlinico Umberto I (ethical code N. 1061bis/2019, 13/09/2019). CACO-2 epithelial cell line from human colon was provided by ATCC (Maryland, USA) and cultured in DMEM supplemented with 10% FBS (Euroclone, UK), L-glutamine, penicillin and streptomycin.

The following antibodies were used: anti-human CD28 (CD28.2, #555726, 2 μg ml^−1^), anti-human CD3 (UCHT1, #555330, 2 μg ml^−1^), goat anti-mouse (GAM, # 553998, 2 μg ml^−1^), anti-human CD3-PE (#555333), anti-human CD45RA BV421 (#562885), mouse anti-human STAT3 (#610189), anti-human CD45RO PE (#555493) (BD Biosciences); rabbit anti-human Lck (#sc-13, 2 μg/ChIP), rabbit anti-human RNA polymerase II (N-20, #sc-899, 2 μg/ChIP), anti-human RelB (C-19, #sc-226, 2 μg/ChIP), anti-human GAPDH (#sc-25778), anti-human mucin 1 (MUC1) (#sc-7313) (Santa Cruz Biotechnology, USA), rabbit anti-human RelA (#8242S) (Cell Signaling Technology, USA), mouse anti-human IL-6 (#MAB206, 10 μg ml^−1^), mouse anti-human IL-22 (#MAB7822, 10 μg ml^−1^) (R&D Systems, USA), anti-human CD4 FITC (#130-114-531, Miltenyi Biotec, Italy). Human recombinant IL-6 (rIL-6) was from Miltenyi Biotec (#130-093-931, 50 ng ml^−1^). The following inhibitory drugs were used: PS1145 (#P6624, Sigma Aldrich), S31-201 (#sc-204304, Santa Cruz Biotechnology), AS605240 (#A0233, Sigma Aldrich).

### CACO-2 Cell Stimulation and Western Blotting

CACO-2 cells were plated at 2.5 × 10^5^ ml^−1^ and co-cultured with CD4^+^ T cells (2 × 10^6^ ml^−1^) in 24 trans-well plates as indicated at 37°C. At the end of incubation, total cell extracts were obtained by lysing cells for 30 min on ice in 1% Nonidet P-40 lysis buffer (150 mM NaCl, 20 mM Tris-HCl (pH 7,5), 1 mM EGTA, 1 mM MgCl_2_, 50 mM NaF, 10 mM Na_4_P_2_O_7_) in the presence of inhibitors of proteases and phosphatases (10 μg ml^−1^ leupeptin, 10 μg ml^−1^ aprotinin, 1 mM NaVO_4_, 1 mM pefablock-SC). Proteins were resolved by SDS-PAGE and blotted onto nitrocellulose membranes. Blots were incubated with the anti-MUC1 or anti-GAPDH (1:400 dilution), extensively washed and after incubation with horseradish peroxidase (HRP)-labeled goat anti-rabbit (#NA934V, 1:5000 dilution) or HRP-labeled goat anti-mouse (#NA931V, 1:5000 dilution) developed with the enhanced chemiluminescence’s detection system (GE Healthcare Life Sciences, Italy). Protein levels were quantified by densitometric analysis using the ImageJ 1.50i program (National Institute of Health, USA).

### ELISA

CD4^+^ T cells were plated at 2 × 10^6^ ml^−1^ in 24-well plate or 24-well plate inserts in the experiments of co-culture with CACO-2 cells (2.5 × 10^5^ ml^−1^) and stimulated for the indicated times with control isotype Abs (Ig) or crosslinked anti-CD28 (2 μg ml^−1^), or anti-CD3 (2 μg ml^−1^) or anti-CD3 plus anti-CD28 Abs (2 μg ml^−1^). Secretion of human IL-22, IL-6, and MMP9 was measured from the supernatants of CD4^+^ T cells cultured alone or with CACO-2 cells and stimulated with control isotype Abs or crosslinked anti-CD28.2 (2 μg ml^−1^), by using human IL-6 (#DY206), IL-22 (#DY782), and MMP9 (#DY911) ELISA kits (R&D Systems). Data were analyzed on a Bio-Plex (Bio-Rad, Hercules, CA, USA). The assays were performed in duplicate. The sensitivity of the assay was 9.4 pg ml^−1^ for IL-6 and 31.2 pg ml^−1^ for IL-22 and MMP9.

### Real-Time PCR

CD4^+^ T cells were plated at 2 × 10^6^ ml^−1^ in 24-well plate or 24-well plate inserts in the experiments of co-culture with CACO-2 cells and stimulated for the indicated times with control isotype matched Abs (Ig) or crosslinked anti-CD28 (2 μg ml^−1^), or anti-CD3 (2 μg ml^−1^) or anti-CD3 plus anti-CD28 Abs (2 μg ml^−1^). Total RNA was extracted by either CD4^+^ T cells or CACO-2 cells using Trizol according to the manufacturer’s instructions and was reverse-transcribed into cDNA by using Moloney murine leukemia virus reverse transcriptase (Thermo Fisher Scientific CA, USA). TaqMan Universal PCR Master Mix and human IL-6, IL-22, MMP9, MUC1, and GAPDH primer/probe sets were from Thermo Fisher Scientific. The relative quantification was performed using the comparative C_T_ method. The results were expressed as arbitrary units (AU) by using the mean value of cells stimulated with control Ig as C_T_ calibrator or fold induction (F.I.) over control Ig-stimulated cells after normalization to GAPDH.

### Plasmids Construction, Cell Transfection, and Cytofluorimetric Analysis

The pIL-22 (-644)-GFP construct containing the GFP under the control of the −644 bp region upstream of the transcriptional start site and the +156 region upstream the translational start site was generated as previously described (36). Briefly, human IL-22 promoter fragment (−1069/+156) was amplified from genomic DNA isolated from human PBMCs by using AmpliTaq (Applied Biosystem). The primers used (excluding additional flanking cloning/restriction sites) were as follow: forward 5′-GGTATTTGCATTTTGATACTTGCATTTGCT-3′ and 5′-TGCAGACAATTCTAACTCGAG-3′. By using an internal AseI restriction site located at position ^−^644 of the human IL-22 promoter, the pIL-22 (-644)-GFP construct was obtained by subcloning the PCR fragment into AseI-Hind III sites within the CMV promoter of pEGFP-N1 vector (Clontech, UK). The sequence of human IL-22 promoter fragment (−644/+156) was verified by DNA sequencing and was as follow: 5′-ATTAATACAATTTTAAGATATATTTACTTCTGCCTTAATTGTTATGATCACTTAAAAATAGTTCCAAAAAGGGAAGAAAACAATAATTAGATTAGCCAAGACAGTTATTTTTGAAACATAAGTCTGGTTTAGAATTCAGCATGTTTAAAAATGAGATAAAATTATTTTAATAATGGAATGATCTGTTAGCTGTCATTACCATTTACTTTAAAGCAGAGGATATAGGACATGGGTCCTTTTTTTCTGATCACCTCCAATGAGATAAGAATCTATAAAGCTGGTAGGAAAATGAGTCCGTGACCAAAATGCTTACTCGGTCACTATAGGAGATCAAAACATTTTATACTAAATCTGAACTCTACTAAGACAAAACAATTGTGTTCTTTGAAAAATATGTAGGGTTTAGAAAATTTCTGGGATTTGTCTGTAAAATACCCTCCGGGCTCTAATAGTGACGTTTTAGGAAAACACTTGCATCTCAAGGTGGAAAGGATAGAGGTGGTGTTTTGTGGGCTCCTGTGGTGGTTAGGTCGTTCTCAGAAGACAGTACTGGAAATTAGATAATTGCTGATGTCATATTTTTCACAATTAAAAAAAAGTCAGTATCCTGGGGGCTATAAAAGCAGCAGCTTCTACCTTCCCCGTCACAAGCAGAATCTTCAGAACAGGTAGGCGTTTCGGCAAACTTGGTACAATTGGTTAGTTTGACGAAATACTTCTTGACTAATTTTGTTCCTTCACGTTGTCTTCGACCAGGTTCTCCTTCCCCAGTCACCAGTTGCTCAAGTTAGAATTGTCTGCA-3′.

Plasmid vectors expressing HA-tagged human RelA, IKKα, IKKβ and NIK were previously described (37, 38).

CD28-positive Jurkat T cells were electroporated (at 260 V, 960 μF) in 0.45 ml RPMI-1640 supplemented with 10% FBS with 1 μg of pIL-22(-644)-GFP together with pcDNA3 control vector 10 μg HA-RelA, or HA-IKKα or HA-IKKβ or 20 μg HA-NIK. Twenty-four hours after transfection, the cells were analyzed by using a BD FACScan flow cytometer (BD Biosciences, Italy). Mean fluorescence intensity was calculated on a total of 5 × 10^3^ GFP-positive events by gating for SSC and FSC.

### Chromatin Immunoprecipitation

10^7^ CD4^+^ T cells were stimulated as indicated and chromatin immunoprecipitation (ChIP) assays were performed as previously described (37). Briefly, after fixing in 1% formaldehyde, T cells were lysed for 5 min in 50 mM Tris, pH 8.0, 2 mM EDTA, 0.1% NP-40, 10% glycerol supplemented with proteases inhibitors. Nuclei were suspended in 50 mM Tris, pH 8.0, 1% SDS, and 5 mM EDTA. Chromatin was sheared by sonication, centrifuged and diluted 10 times in 50 mM Tris, pH 8.0, 0.5% NP-40, 0.2 M NaCl, 0.5 mM EDTA. After pre-clearing with a 50% suspension salmon sperm–saturated Protein-A or Protein G Sepharose beads (Amersham), lysates were incubated at 4°C overnight with anti-RelA (1:100 dilution), anti-RelB (2 μg), anti phoshoTyr705 STAT3 (pSTAT3, 1:100 dilution), anti-RNA-polymerase II (Pol II, 2 μg), or control anti-Lck Abs (2 μg). Immune complexes were collected with sperm–saturated Protein-A or Protein G Sepharose beads, washed three times with high salt buffer (20 mM Tris, pH 8.0, 0.1% SDS, 1% NP-40, 2 mM EDTA, 500 mM NaCl), and five times with 1× Tris/EDTA (TE). Immune complexes were extracted in 1× TE containing 1% SDS, and protein–DNA cross-links were reverted by heating at 65°C overnight. DNA was extracted by phenol–chloroform and about 1/30 of the immunoprecipitated DNA was analyzed by real-time PCR. Quantitative real-time PCR with SensiMix™ SYBR^®^ Hi-ROX Kit (Bioline, UK) was performed for the human IL-22 promoter. Specific enrichment was calculated as previously described (39) by using the cycle threshold (Ct): 2^(Ct of control ChIP-Ct of control Input)/^2^(Ct of specific ChIP - Ct of specific Input)^. The human IL-22 promoter primers used for each specific ChIP were as follow: RelA I, RelB I and pSTAT3 I, 5′-GCTTACTCAGCCACTATAGGAGATCA-3′ and 5′-CCGGAGGGTATTTTACAGACAAATCC-3′; RelA II, 5′-ACCCTCCGGGCTCTAATAGTGAC-3′ and 5′-AGAACGACCTAACCACCACAGGA-3′; pSTAT3 II and Pol II 5′-CCTGTGGTGGTTAGGTCGTTCT-3′ and 5′-GCTGCTTTTATAGCCCCCAGGAT-3′.

### Cytotoxicity Assay

The cytotoxicity of AS605240 on CACO-2 cells was evaluated by propidium iodide (PI) staining (10 μg ml^−1^). CACO-2 cells were plated at 2.5 × 10^5^ cells ml^−1^ in 24-well plates and treated with 10 μM AS605240 or DMSO, as vehicle control, for 48 h. Cytotoxicity was analyzed by a BD Biosciences FACScalibur (Mountain View, CA) by quantifying the percentage of PI positive cells. Results were calculated from at least three independent experiments.

### Statistical Analysis

The sample size was chosen based on previous studies to ensure adequate power. Parametrical statistical analysis (mean and SEM) was performed to evaluate differences between continuous variables through Prism 5.0 (Graph Pad Software, San Diego, CA) using Student t test. For multiple group comparisons, significant differences were calculated using nonparametric Mann–Whitney *U* or Wilcoxon tests, and linear regression analyses were performed using the Pearson chi-squared test. For all tests, p values < 0.05 were considered significant.

## Results

### CD28 Autonomous Stimulation Up-Regulates IL-22 Gene Expression and Secretion in Human CD4^+^ T Cells

**Figure 1:**
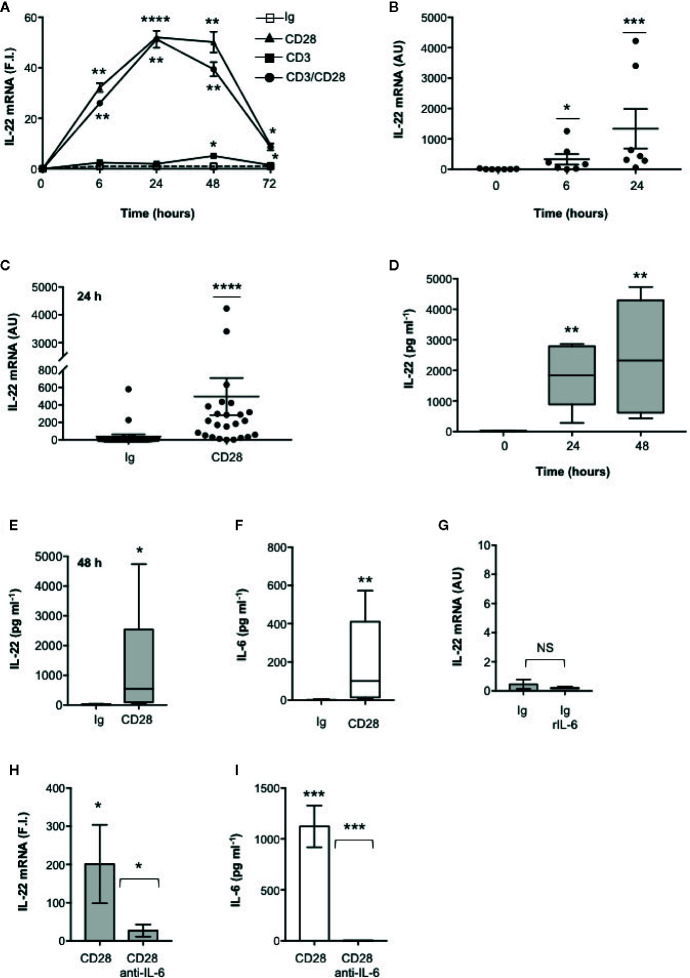
IL-22 regulation by CD28 autonomous stimulation in human CD4^+^ T cells. **(A)** CD4^+^ T cells were stimulated for indicated times with 2 μg ml^−1^ isotype control (Ig) or anti-CD28.2 or anti-CD3 (UCHT1) or anti-CD3 plus anti-CD28.2 Abs. IL-22 mRNA expression was measured by real-time PCR and values, normalized to GAPDH, expressed as fold induction (F.I.) over the basal level of cells stimulated with isotype control Ig. Data show the mean ± SEM of one out of three HD subjects. Statistical significance was calculated by Student t test. **(B)** IL-22 mRNA levels of CD4^+^ T cells from HD (n = 7) stimulated for 0, 6, or 24 h with crosslinked anti-CD28.2 Abs. IL-22 mRNA levels were measured by real-time PCR and values, normalized to GAPDH, expressed as arbitrary units (AU). Data show the mean ± SEM and statistical significance was calculated by Mann-Whitney test. Mean values: 0 h = 8.5, 6 h = 329.6, 24 h = 1336. **(C)** IL-22 mRNA levels (AU) of CD4^+^ T cells from HD (n = 24) stimulated for 24 h with isotype control Ig or anti-CD28.2 Abs. Data show the mean ± SEM and statistical significance was calculated by Mann-Whitney test. Mean values: Ig = 38.1; CD28 = 496.7. **(D)** CD4^+^ T cells from HD (n = 6) were stimulated for 0, 24 or 48 h with isotype control or crosslinked anti-CD28.2 Abs. IL-22 levels in culture supernatant were measured by ELISA. Lines represent median values and statistical significance was calculated by Student t test. Median values: 0 h = 17.7 pg ml^−1^, 24 h = 1840 pg ml^−1^, 48 h = 2329 pg ml^−1^. **(E)** CD4^+^ T cells from HD (n = 12) were stimulated for 48 h with isotype control Ig or anti-CD28.2 Abs. IL-22 levels in culture supernatant were measured by ELISA. Lines represent median values and statistical significance was calculated by Student t test. Median values: Ig = 14.4 pg ml^−1^, CD28 = 545 pg ml^−1^. **(F)** CD4^+^ T cells from HD (n = 8) were stimulated for 24 h with isotype control Ig or anti-CD28.2 Abs. IL-6 levels in culture supernatant were measured by ELISA. Lines represent median values and statistical significance was calculated by Student t test. Median values: Ig = 0 pg ml^−1^, CD28 = 99.3 pg ml^−1^. **(G)** CD4^+^ T cells from HD (n = 3) were stimulated for 24 h with isotype control Ig in the presence or absence of 50 ng ml^−1^ recombinant IL-6 (rIL-6). IL-22 mRNA levels were measured by real-time PCR and values, normalized to GAPDH, expressed as arbitrary units (AU). Data show the mean ± SEM and statistical significance was calculated by Student t test. **(H, I)** IL-22 mRNA expression **(H)** and secretion **(I)** in CD4^+^ T cells from HD stimulated for 24 h **(H)** or 48 **(I)** with isotype control Ig or anti-CD28.2 Abs in the presence of 10 μg ml^−1^ isotype control or neutralizing anti-IL-6 Abs. IL-22 mRNA levels **(H)** were measured by real-time PCR and values (n = 7), normalized to GAPDH, expressed as fold induction (F.I.) over the basal level of cells stimulated with isotype control Ig. Data show the mean ± SEM and statistical significance was calculated by Wilcoxon test. Mean values: CD28 = 201.3, CD28 anti-IL-6 = 25.7. IL-22 levels **(I)** in culture supernatants were measured by ELISA (n = 6). Data show the mean ± SEM and statistical significance was calculated by Student t test. Mean values: Ig = 0 pg ml^−1^, CD28 = 1123 pg ml^−1^, CD28 anti-IL-6 = 0 pg ml^−1^. (*) p < 0.05, (**) p < 0.01, (***) p < 0.001, (****) p < 0.0001. NS, not significant.

**Figure 2:**
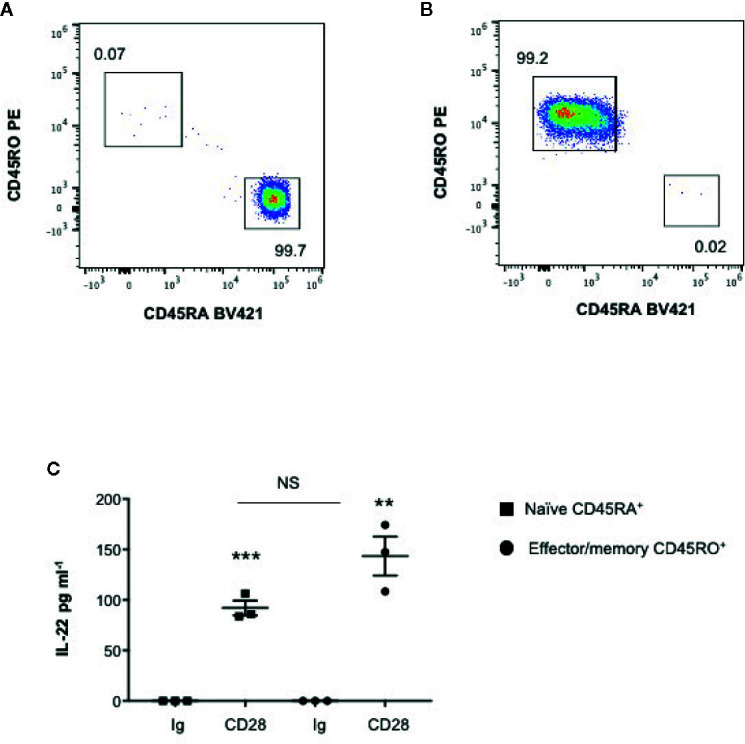
CD28 induces up-regulation of IL-22 gene secretion in both naïve CD45RA^+^ and effector/memory CD45RO^+^ CD4^+^ T cells. **(A, B)** FACS analysis of naïve CD45RA **(A)** and effector/memory CD45RO **(B)** sorted CD4^+^ T cells. **(C)** IL-22 secretion in culture supernatants measured by ELISA of naïve (CD45RA) or effector/memory (CD45RO) CD4^+^ T cells from HD (n = 3) stimulated for 48 h with isotype control Ig or anti-CD28.2 Abs. Data show the mean ± SEM and statistical significance was calculated by Student t test. Mean values: CD45RA Ig = 0, CD45RA CD28 = 92; CD45RO Ig = 0, CD45RO CD28 = 143.4. (**) p < 0.01, (***) p < 0.001. NS, not significant.

We have recently found that CD28 stimulation induces the expression of Th17 related cytokines in CD4^+^ T cells from HD, relapsing-remitting MS (RRMS) and T1D patients (32–34). More recent preliminary data also evidenced that CD28 stimulation up-regulated IL-22 gene expression in peripheral CD4^+^ T cells from stable RRMS patients (35). In order to verify whether CD28-induced IL-22 expression was also effective in HD and clarify the molecular basis of this activity, we performed a kinetic analysis of IL-22 gene expression by stimulating human CD4^+^ T cells from HD with an agonistic anti-CD28 Ab (CD28.2) binding the same epitope recognized by B7 molecules (40) or anti-CD3 (UCHT1) or anti-CD3 plus anti-CD28 Abs. CD28 stimulation of CD4^+^ T cells from one representative HD induced a significant increase (p < 0.01) of IL-22 gene expression within 6 h that further increased 24 to 48 h and decreased 72 h after stimulation (**Figure 1A**). No significant differences in neither IL-22 mRNA levels nor kinetics were observed between CD28 and CD3 plus CD28 stimulation, whereas CD3 stimulation alone induced only a slight increase of IL-22 gene expression after 48 h (**Figure 1A**). Similar results were obtained by analyzing IL-22 gene expression in CD28-stimulated CD4^+^ T cells from a larger sample size (n = 7) (**Figures 1B, C**). CD28-induced IL-22 gene expression was also associated with a strong increase of IL-22 cytokine secretion after 24 to 48 h from stimulation (**Figures 1D, E**). In the human system, IL-22 gene expression has been described to be induced by IL-6 (41). Consistently with our previous data (33), CD28 stimulation induced a strong IL-6 secretion (**Figure 1F**). IL-6–mediated signaling alone was not sufficient for inducing IL-22 gene expression (**Figure 1G**). However, the blockade of IL-6–mediated signaling by a neutralizing anti–IL-6 Ab strongly inhibited CD28-induced IL-22 gene expression (**Figure 1H**) and secretion (**Figure 1I**) in CD4^+^ T cells from HD. As we previously observed for IL-17A (33) and other pro-inflammatory cytokines (31), the analysis of CD28-induced IL-22 secretion in purified naïve CD4^+^CD45RA^+^ (**Figure 2A**) or effector/memory CD4^+^CD45RO^+^ (**Figure 2B**) evidenced no significant differences between the two population (**Figure 2C**).

These data evidence that CD28 intrinsic signaling regulates IL-22 gene expression and secretion in both naïve and effector/memory CD4^+^ T cells in a IL-6–dependent manner.

### STAT3 and RelA/NF-κB Transcription Factors Regulate CD28-mediated IL-22 Gene Expression

**Figure 3:**
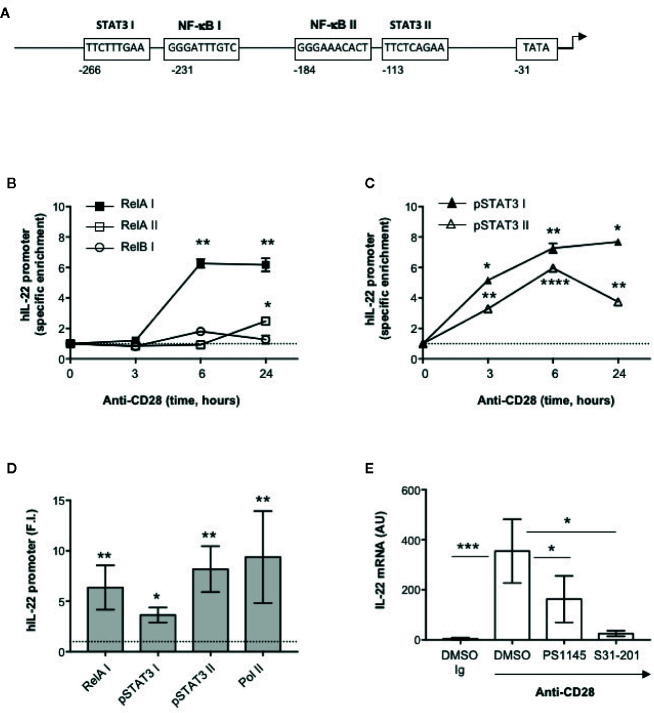
Human IL-22 promoter regulation by RelA and pSTAT3 in CD28-stimulated T cells. **(A)** Schematic organization of the region upstream the transcriptional start point of human IL-22 promoter with positions and sequences of the binding sites for NF-κB (NF-κB I and NF-κB II), STAT3 (STAT3 I and STAT3 II) and TATA box. **(B, C)** CD4^+^ T cells were stimulated for the indicated times with anti-CD28.2 Abs and anti-RelA, anti-RelB **(B)** anti-pSTAT3 **(C)** ChIPs were performed. Immunoprecipitated DNA was analyzed by real time PCR by using IL-22 promoter specific primers covering the NF-κB I and II (RelA I, RelA II and RelB I) and STAT3 I and II binding sites. Specific enrichment over isotype control Abs was calculated by the Cτ method. Data express the mean ± SEM of one out of three HD. Significance was calculated by Student t test. **(D)** Real time PCR of IL-22 promoter regions NF-κB I, STAT3 I and II, and TATA Box from anti-pSTAT3, anti-RelA and anti-RNA polymerase II (Pol II) ChIPs performed on CD4^+^ T cells from HD (n = 9) stimulated for 24 h with isotype control Ig or anti-CD28.2 Abs. Specific enrichment over negative control Abs (anti-Lck) Abs was calculated by the Cτ method and data expressed as fold inductions (F.I.) over isotype control Ig-stimulated cells. Data express the mean ± SEM and significance was calculated by Student t test. Mean values: RelA I = 6.5, STAT3 I = 3.6, STAT3 II = 7.8, Pol II = 11.6. **(E)** IL-22 mRNA levels (AU) of CD4^+^ T cells from HD (n = 5) treated with DMSO, as vehicle control, or 10 μM PS1145 or 100 μM S31-201 and stimulated for 24 h with isotype control Ig or anti-CD28.2 Abs. Data express the mean ± SEM and statistical significance was calculated by Mann-Whitney test. Mean values: DMSO Ig = 4.5, DMSO CD28 = 641.5, PS1145 CD28 = 163.2, S31-201 CD28 = 25.4. (*) p < 0.05, (**) p < 0.01, (***) p < 0.001, (****) p < 0.0001.

The human IL-22 gene promoter contains two putative STAT3 responsive elements (STAT3 I and II) and two NF-κB binding sites (NF-κB I and II) upstream of the transcription start site that have been involved in IL-22 promoter trans-activation (42, 43) (**Figure 3A**). We have previously demonstrated that human CD28 individual stimulation induces the activation and nuclear translocation of RelA (31, 44) and, more recently, we evidenced that CD28 induces the phosphorylation on Tyr705 of STAT3 (pSTAT3) and its nuclear translocation in a IL-6–dependent manner. pSTAT3 and RelA/NF-κB in turn cooperate for inducing the promoter trans-activation and transcription of IL-17A (33). To assess if pSTAT3 and RelA were also involved in CD28-mediated up-regulation of IL-22, we performed chromatin immunoprecipitation (ChIP) assays in peripheral CD4^+^ T cells stimulated with agonistic anti-CD28.2 Abs. To do that, three distinct oligonucleotide probes were used; probe I (−338 to −203) for both STAT3 I and NF-κB I binding sites, probe II (−213 to −110) for NF-κB II binding site and probe III (−131 to −20) for both STAT3 II binding site and TATA box (**Figure 3A**). The kinetic analysis of the specific recruitment to the IL-22 promoter evidenced that RelA efficiently bound NF-κB I but not NF-κB II consensus sequences within 6 h from stimulation and persisted over 24 h, whereas no significant recruitment of RelB was observed (**Figure 3B**). pSTAT3 was significantly recruited on both STAT3 I and STAT3 II consensus sequences on the IL-22 promoter with similar kinetics (**Figure 3C**). The specific recruitment of both RelA on the NF-κB I and pSTAT3 on both I and II binding sites was confirmed in CD4^+^ T cells from a larger sample size (n = 9) and was associated to CD28-induced transcriptional activation of IL-22 promoter, as evidenced by RNA polymerase II (pol II) promoter occupancy (**Figure 3D**). The significant inhibition of IL-22 gene expression exerted by the STAT3 inhibitor S31-201 (45, 46) and the NF-κB inhibitor PS1145 (47) (**Figure 3E**), confirmed the mutual cooperation of both transcription factors in regulating CD28-induced IL-22 expression.

### Functional Relevance of CD28-Mediated IL-22 Production in Epithelial Cell Functions

**Figure 4:**
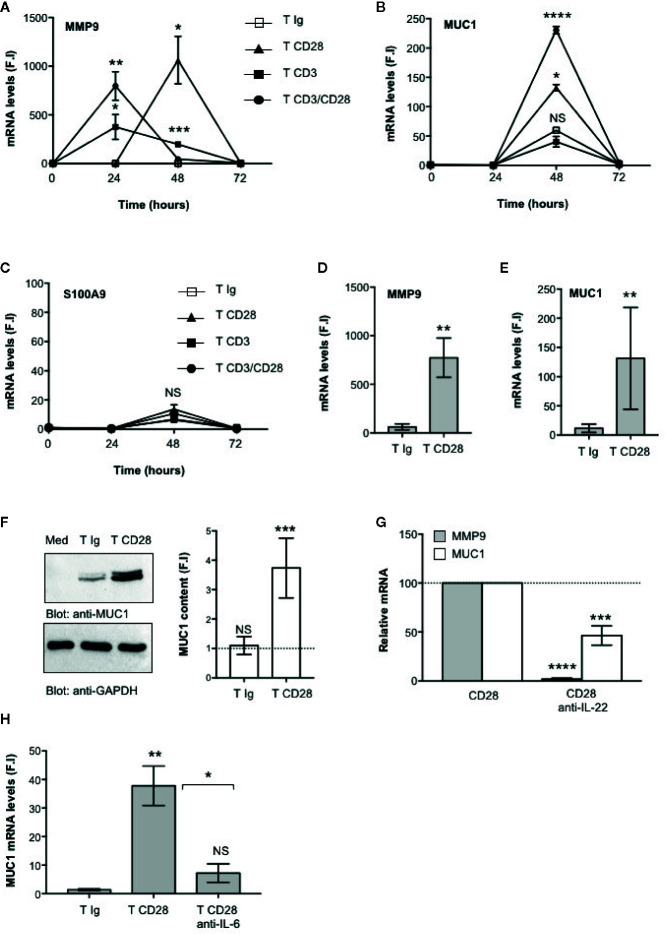
CD28-mediated IL-22 production by CD4^+^ T cells induces the up-regulation of MUC1 and MMP9 in CACO-2 epithelial cells. **(A–C)** CACO-2 cells were cultured in triplicates in 24 trans-well plates with medium alone or CD4^+^ T cells stimulated for the indicated times with isotype control Ig or crosslinked anti-CD28.2 or anti-CD3 (UCHT1) or anti-CD3 plus anti-CD28.2 Abs. MMP9 **(A)**, MUC1 **(B)** and S100A9 **(C)** mRNA levels in CACO-2 cells were measured by real-time PCR and values, normalized to GAPDH, expressed as fold inductions (F.I.) over the basal level of CACO-2 cultured with medium alone. Data show the mean ± SEM of one out of three HD. Statistical significance was calculated by Student t test. **(D, E)** MMP9 **(D)** and MUC1 **(E)** mRNA levels in CACO-2 cells cultured with medium alone or with CD4^+^ T cells from HD (n = 8) stimulated for 48 h with isotype control Ig or cross-linked anti-CD28.2 Abs. Values, normalized to GAPDH, were expressed as fold inductions (F.I.) over the basal level of CACO-2 cultured with medium alone. Data show the mean ± SEM and statistical significance was calculated by Student t test. Mean values: MMP9, T Ig = 62.4, T CD28 = 773.4; MUC1, T Ig = 11.5, T CD28 = 131.1. **(F)** Anti- MUC1 or anti- GAPDH western blotting of CACO-2 cells cultured with medium alone (Med) or co-cultured with CD4^+^ T cells stimulated for 48 h with isotype control Ig or cross-linked anti-CD28.2 Abs. MUC1 fold inductions (F.I.) were quantified by densitometric analysis and normalized to GAPDH levels. Bars represent mean F.I. ± SEM of five HD. **(G)** CACO-2 cells were cultured with medium alone or co-cultured with CD4^+^ T cells from HD (n = 5) stimulated for 48 h with isotype control Ig or anti-CD28.2 Abs in the presence of 10 μg ml^−1^ isotype control or neutralizing anti-IL-22 Abs. MMP9 and MUC1 mRNA levels were analyzed by real time PCR and, after normalization to GAPDH, fold inductions (F.I.) were calculated over the basal level of CACO-2 cultured with medium alone. Values of CACO-2 cells co-cultured with CD4^+^ T cells treated with DMSO and stimulated with anti-CD28 Abs were assumed as 100%. Data express the mean ± SEM. Statistical significance was calculated by Student t test. **(H)** CACO-2 cells were cultured with medium alone or co-cultured with CD4^+^ T cells from HD (n = 3) stimulated for 48 h with isotype control Ig or anti-CD28.2 Abs in the presence of 10 μg ml^−1^ isotype control or neutralizing anti-IL-6 Abs. MUC1 mRNA levels were analyzed by real time PCR and, after normalization to GAPDH, fold inductions (F.I.) were calculated over the basal level of CACO-2 cultured with medium alone. Data express the mean ± SEM. Statistical significance was calculated by Student t test. Mean values: T Ig = 1.3, T CD28 = 37.8, T CD28 anti-IL-6 = 7.2. (*) p < 0.05, (**) p < 0.01, (***) p < 0.001, (****) p < 0.0001, NS, not significant.

The main functional effects of IL-22 are exerted on epithelial cells, such as intestinal cells, where IL-22 promotes the production of anti-microbial peptides, mucins and matrix metalloproteases (MMPs) required for epithelial barrier functions and protection against extracellular pathogens (8, 9). We next analyzed the functional role of CD28-mediated activation of IL-22–producing T cells on intestinal epithelial cells. To do that, CD4^+^ T cells were stimulated with control isotype matched Abs (Ig), or anti-CD28, or anti-CD3 or anti-CD3 plus anti-CD28 Abs and co-cultured in trans-well plates with CACO-2 cells for different times. The gene expressions of mucin 1 (MUC1), MMP9 and S100A9 anti-microbial peptide were then analyzed. The kinetic analysis evidenced that both MMP9 (**Figure 4A**) and MUC1 (**Figure 4B**) gene expressions were strongly up-regulated in CACO-2 cells after 48 h of co-culture with CD28-stimulated CD4^+^ T cells, whereas no significant increase in S100A9 was observed (**Figure 4C**). Similar results were obtained by analyzing MMP9 (**Figure 4D**) and MUC1 (**Figure 4E**) gene expression in CACO-2 cells co-cultured with CD28-stimulated CD4^+^ T cells from a larger sample size (n = 9). Moreover, the up-regulation and MUC1 gene expression induced by co-culturing CACO-2 cells with CD28-stimulated CD4^+^ T cells was also associated with a strong increase of MUC1 protein content (**Figure 4F**) after 48 h from stimulation. In contrast to the gene expression data (**Figure 4D**), when we looked at MMP9 secretion we found high levels of MMP9 in culture supernatants from CACO-2 cells co-cultured with CD4^+^ T cells stimulated with control Ig that further increased following CD28 stimulation (**Supplementary Figure S1A**), thus suggesting that CD4^+^ T cells also produced MMP9, as previously reported (48). CD4^+^ T cells secreted, indeed, high levels of MMP9 in culture supernatant and no significant differences were observed following CD28 stimulation (**Supplementary Figure S1B**). The significant inhibition of MMP9 and MUC1 gene expressions mediated by anti-IL-22 neutralizing Abs (**Figure 4G**) strongly support a role of IL-22 in CD28-induced up-regulation of MMP9 and MUC1 in CACO-2 cells. Consistently with the pivotal role of IL-6–mediated signaling in regulating both IL-22 transcription (**Figure 1H**) and secretion (**Figure 1I**), MUC1 gene expression was strongly impaired by anti–IL-6 neutralizing Abs in CACO-2 cells co-cultured with CD28-stimulated CD4^+^ T cells (**Figure 4H**).

### CD28-Associated Class 1A PI3K Regulates IL-22 Expression and IL-22–Mediated Up-Regulation of MMP9 and MUC1 in CACO-2 Cells

**Figure 5:**
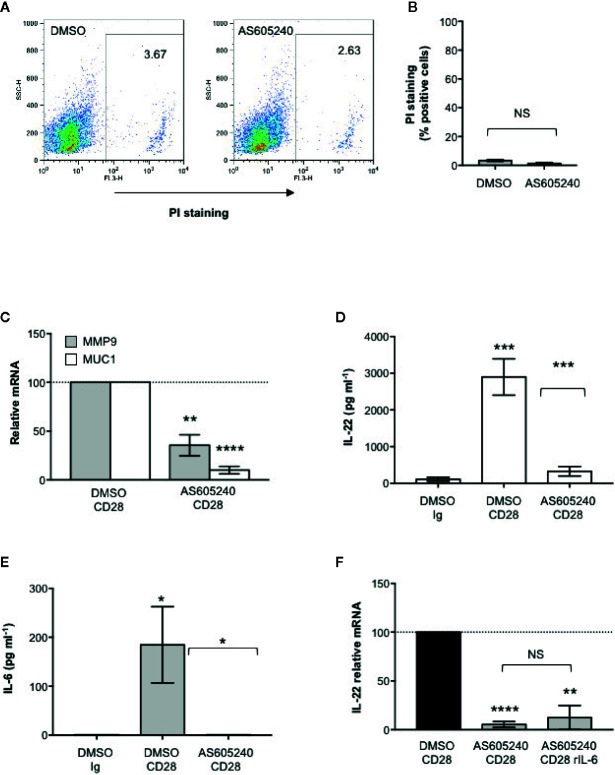
CD28-associated class 1A PI3K regulates IL-22 production and IL-22–mediated epithelial cell functions. **(A)** CACO-2 cells were cultured for 48 h with DMSO, as vehicle control, or 10 μM AS605240. Cell death was analyzed by flow cytometry by quantifying the ability of cells to incorporate propidium iodide (PI). **(B)** The percentage of PI positive cells was calculated. Results express the mean ± SEM and statistical significance was calculated by Student t test. **(C)** MMP9 and MUC1 mRNA levels in CACO-2 cells cultured with medium alone or CD4^+^ T cells from HD (n = 3) treated with DMSO, as vehicle control, or 10 μM AS605240 and stimulated for 48 h with isotype control Ig or anti-CD28.2 Abs. mRNA levels were analyzed by real-time PCR and, after normalization to GAPDH, fold inductions (F.I.) were calculated over the basal level of CACO-2 cultured with medium alone. Values of CACO-2 cells co-cultured with CD4^+^ T cells treated with DMSO and stimulated with anti-CD28 Abs were assumed as 100%. Data express the mean ± SEM. Statistical significance was calculated by Student t test. **(D)** IL-22 levels in culture supernatant, measured by ELISA, of CD4^+^ T cells from HD (n = 7) treated with DMSO, as vehicle control, or 10 μM AS605240 and stimulated for 48 h with isotype control Ig or crosslinked anti-CD28.2 Abs. Data express mean ± SEM and statistical significance was calculated by Student t test. Mean values: DMSO Ig = 112.1, DMSO CD28 = 2897, AS605240 CD28 = 328.5. **(E)** IL-6 levels in culture supernatant, measured by ELISA, of CD4^+^ T cells from HD (n = 7) treated with DMSO, as vehicle control, or 10 μM AS605240 and stimulated for 24 h with isotype control Ig or crosslinked anti-CD28.2 Abs. Data express mean ± SEM and statistical significance was calculated by Student t test. Mean values: DMSO Ig = 0 pg ml^−1^, DMSO CD28 = 184.7 pg ml^−1^, AS605240 CD28 = 0 pg ml^−1^. **(F)** IL-22 mRNA levels in CD4^+^ T cells from HD (n = 3) treated with DMSO, as vehicle control, or 10 μM AS605240 and stimulated for 24 h with isotype control Ig or anti-CD28.2 Abs. Fold inductions (F.I.) were calculated over the basal level of DMSO-treated T cells stimulated with isotype control Ig. Values of CD4^+^ T cells treated with DMSO and stimulated with anti-CD28 Abs were assumed as 100%. Data express the mean ± SEM and statistical significance was calculated by Student t test. (*) p < 0.05), (**) p < 0.01, (***) p < 0.001, (****) p < 0.0001. NS, not significant.

CD28 pro-inflammatory functions depend on the ability of its intracytoplasmic tail to recruit and activate class 1A PI3K (49–51). We have recently demonstrated that class 1A PI3K activity is crucial for CD28-induced up-regulation of pro-inflammatory cytokines related to Th17 cell phenotype in both HD and RRMS patients (33, 35). Consistently, a non-cytotoxic dose of the class 1A PI3K inhibitor AS605240 (52) (**Figures 5A, B**), significantly impaired MMP9 and MUC1 gene expression in CACO-2 cells co-cultured with CD28-stimulated CD4^+^ T cells (**Figure 5C**) as well as CD28-induced IL IL-22 (**Figure 5D**) and IL-6 secretion (**Figure 5E**). Moreover, the inability of recombinant IL-6 to restore impaired IL-22 gene expression in AS605240-treated CD28-stimulated CD4^+^ T cells, strongly support a role of class 1A PI3K downstream CD28.

Altogether these data support a crucial role of CD28-associated PI3K in IL-22–regulated epithelial barrier functions.

## Discussion

IL-22 is an important cytokine that strengthen epithelial barrier functions against extracellular pathogen and regulates tissue homeostasis and repair. However, excessive production of IL-22 has been also associated with severe complications in several pathogen infections (12), including the recently identified Sars-Cov2 (18) as well as with inflammatory bowel diseases such as Crohn’s disease (23), neurological disorders such as MS and Guillain-Barrè syndrome (25, 26), and rheumatoid arthritis (53, 54). Therefore, the identification of receptors and associated signaling mediators regulating IL-22 expression could represent an important goal of the ongoing research in inflammation and autoimmunity.

In mouse T cells, IL-22 is mainly regarded as a Th17 cytokine (41, 55, 56), whereas in human CD4^+^ T cells IL-22 expression often does not correlate with the expression of IL-17 or the master transcriptional regulator of Th17 cells retinoic acid receptor-related orphan nuclear receptor γt (RORγt) (57, 58) and is produced by a unique memory Th22 cell subset (3, 4). Herewith, we evidence that CD28 stimulation induced IL-22 expression and production in the absence of TCR engagement (**Figure 1**). The up-regulation of IL-22 transcription by CD28 autonomous stimulation of peripheral CD4^+^ T cell followed a kinetic (**Figures 1A, B**) similar to IL-17A (33) and no significant differences were observed between naïve (CD45RA) and effector/memory (CD45RO) CD4^+^ T cells (**Figure 2**). Moreover, we found that CD28-induced up-regulation of IL-22 depended on CD28-induced IL-6 (**Figure 1F**), as demonstrated by the strong inhibitory effects exerted by neutralizing anti-IL-6 Abs (**Figures 1H, I**). However, although IL-6 itself is able to prime IL-22 production in activated human CD4^+^ T cells (41), stimulation of CD4^+^ T cells with IL-6 alone is not sufficient for up-regulating IL-22 gene expression (**Figure 1G**) but requires a second signal delivered by CD28. In this contest, among the most promising therapeutic strategies for dampening excessive inflammatory responses and ARDS in SARS-Cov2 infection, tocilizumab, an anti–IL-6R mAb inhibitor that interferes with IL-6–mediated signaling and has already approved for RA treatment (59, 60) resulted effective in preventing the cytokine storm, ARDS and to reduce mortality in COVID-19 patients (61–63).

IL-6 regulates the expression of both IL-17A and IL-22 through IL-6 receptor-associated STAT3 (41, 55, 64–67). We have recently demonstrated that CD28 stimulation induced a strong and persistent STAT3 phosphorylation on Tyr705 and its nuclear translocation in CD4^+^ T cells. We also found that activated STAT3 was also recruited to the proximal human IL-17A promoter and induced its trans-activation in response to CD28 stimulation (33). In T cells from STAT3 deficient mice, IL-22 expression is impaired (42, 68), whereas the overexpression of constitutively active STAT3 up-regulates IL-22 expression (66). For instance, two functional STAT3 binding sites have been identified upstream of the transcription start site within the mouse IL-22 promoter (42). The human IL-22 promoter contains similar high consensus sequences (**Figure 3A**) and we found that CD28 stimulation promoted the recruitment of STAT3 on both binding sites in human CD4^+^ T cells (**Figures 3C, D**). These data together with the strong inhibition of CD28-induced IL-22 expression by the STAT3 inhibitor S31-201 (**Figure 3E**), support a direct role of STAT3 in regulating IL-22 gene expression in CD28-stimulated CD4^+^ T cells.

In the human IL-22 promoter, two putative NF-κB responsive elements (43), NF-κB I and II (**Figure 2A**), containing the consensus binding sequence GGGRNNYYCC (where R represents A or G, N represents any nucleotide, and Y represents C or T) has been also identified (69). The NF-κB family comprises five members: p50/NF-κB1, p52/NF-κB2, RelA/p65, RelB and c-Rel. RelA, c-Rel and RelB contain a transactivation domain and form transcriptionally active heterodimers in association with p50 and/or p52 (70). We have demonstrated that CD28 autonomous stimulation by either B7.1/CD80 or anti-CD28.2 agonistic Abs activates a non-canonical NF-κB2-like cascade (27) by recruiting and activating NIK and IKKα (71, 72), thus leading to the nuclear translocation of RelA-containing NF-κB dimers and to the trans-activation of pro-inflammatory target cytokines, including IL-17A (31–33, 44). Consistently, RelA overexpression strongly up-regulated human IL-22 promoter-GFP construct [hIL-22 prom(-644)-GFP], containing the GFP construct under the control of the IL-22 5′-flanking region (−644) upstream of the transcriptional start point (**Supplementary Figure S2**). Moreover, as observed in ILC3 cells (43), RelA/NF-κB binds the high homology consensus sequence II at position −184 of the human IL-22 promoter (**Figure 3A**) and cooperates with STAT3 for inducing IL-22 transcription in CD28-stimulated CD4^+^ T cells (**Figures 3B, D**). Although both IKKα and IKKβ induced a significant (p < 0.05) trans-activation of hIL-22 promoter at similar levels (1.6 fold inductions) when overexpressed in Jurkat cells, a strongest up-regulation (4 fold inductions) of hIL-22 promoter was induced by NIK overexpression (**Supplementary Figures S1D, E**). These data are consistent with those from Rudloff et al., who identified IKKα as an important regulator of IL-22 gene expression (36).

IL-22 regulates essential functions in host defense against pathogens by mediating the crosstalk between the immune system and epithelial cells. In fact, IL-22 regulates the expression of several genes that maintain the barrier functions of intact epithelium (9). These include molecules involved in tight and gap junctions such as claudins that keep epithelial cells together and regulate the permeability of intestinal epithelial barrier to water, nutrients and ions (73), anti-microbial proteins such as S100A (55, 74) and mucins. MUC1, a membrane-bound mucin, is the main component of the intestinal mucus layer that provides a physiological barrier to prevent close contacts between epithelial cells and microbes (75). In addition to its physiological barrier functions, MUC1 has been described to modulate the functions of inflammatory T cells. In MUC1-deficient mice, Th17 cells and IL-17–producing ILC3 cells were expanded and exacerbated colitis (76). Similarly, a reduced glycosylation of MUC1 increased both susceptibility to colitis and progression to colon cancers (77, 78). In both human and mouse colonic epithelial cell lines, IL-22 has been reported to promote MUC1 expression (79, 80). We extend these data by evidencing an important role of CD28 in regulating epithelial barrier functions. During CD28 stimulation, RelA/NF-κB and IL-6–associated STAT3 cooperate for inducing IL-22 expression and secretion (**Figure 3**) that in turn acts on epithelial cells by promoting the up-regulation of MUC1 (**Figure 4**). Moreover, IL-22 secreted by CD28-activated CD4^+^ T cells also stimulate MMP9 expression in CACO-2 cells (**Figure 4**). Interestingly, Pujada et al. recently showed that MMP9 expression in colonic epithelium was associated with a more efficient epithelial barrier functions by maintaining epithelial integrity, microbiota and immune homeostasis (81). However, MMP9 is also up-regulated in inflamed intestine of IBD patients and has been recently suggested as a predictor marker of disease exacerbation in CD (82, 83). Moreover, high levels of MMP9 were also found in the serum and cerebrospinal fluid of MS patients and increase during relapses (84, 85). Thus, anti-inflammatory compounds able to dampen excessive production of MMPs might represent potential therapeutic strategies for inflammatory diseases. Our findings on the ability of class 1A PI3K inhibitor to impair both CD28-induced IL-6 and IL-22 production as well as IL-22–dependent MMP9 and MUC1 expression in CACO-2 cells (**Figure 5**) are consistent with data from Yan et al. who identified the PI3K/Akt/mTORC1 as a crucial pathway in regulating both Th17 cell-mediated inflammation and lung injury in ARDS (15).

Our results provide novel insights on the role of CD28 and associated signaling mediators in IL-22 regulation in human CD4^+^ T cells and may provide the biological bases for the development of new therapeutic strategies to dampen inflammatory and autoimmune disorders.

## Data Availability Statement

The raw data supporting the conclusions of this article will be made available by the authors, without undue reservation.

## Ethics Statement

The studies involving human participants were reviewed and approved by Ethical Committee of the Policlinico Umberto I (ethical code N. 1061bis/2019, 13/09/2019). The patients/participants provided their written informed consent to participate in this study.

## Author Contributions

MK performed most of the experiments, analyzed the data, interpreted the results, and helped in writing the manuscript. CA, SF, SC, MS, and MB performed parts of the experiments and data analyses. SA contributed with human samples for the study. MK, CA, SF, MS, and LB were involved in the discussion about the data. LT designed the study, coordinated the work, and wrote the manuscript. All authors contributed to the article and approved the submitted version.

## Funding

This work was supported by: the Italian Foundation for Multiple Sclerosis (FISM 2016/R/29), “Progetto Ateneo” (Sapienza University of Rome, Italy) and Istituto Pasteur Italia-Fondazione Cenci Bolognetti (Sapienza University of Rome, Italy) to LT; the Italian Ministry of Health (Progetto di ricerca Finalizzata RF-2018-12366111) and the Italian Foundation for Multiple Sclerosis (FISM Progetto Speciale 2018/S/5) to LB; the Italian Ministry of Health (GR-2016-02363725 and GR-2018- 12365529) to MS.

## Conflict of Interest

The authors declare that the research was conducted in the absence of any commercial or financial relationships that could be construed as a potential conflict of interest.
