# Supplementary material for: CD28 Autonomous Signaling Orchestrates IL-22 Expression and IL-22-Regulated Epithelial Barrier Functions in Human T Lymphocytes
Source: Front Immunol. 2020 Oct 14;11:590964. doi: 10.3389/fimmu.2020.590964 (PMC7592429; doi:10.3389/fimmu.2020.590964)
Supplement: Supplementary file 1 [file DataSheet_1.pdf]

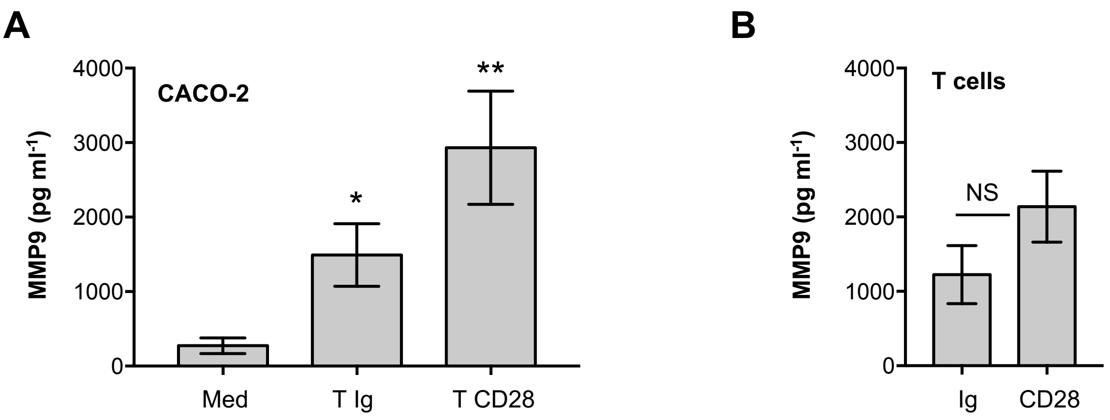

Figure S1. **MMP9 secretion in CD28-stimulated CD4+ T cells.** (**A**) MMP9 levels in supernatants of CACO-2 cells cultured for 48 h in trans-well plates with medium alone (Med) or with $CD4^+$ T cells from HD subjects (n = 11) stimulated with isotype control Ig or crosslinked anti-CD28.2 Abs. Bars show the mean ± SEM and statistical significance was calculated by Student t test. (**B**) MMP9 secretion in culture supernatants of $CD4^+$ T cells from HD subjects (n = 8) stimulated for 48 h with isotype control or anti-CD28.2 Abs. The secretion was measured by ELISA. Lines represent mean ± SEM. *$p < 0.05$, **$p < 0.01$, NS=not significant.
