# Supplementary material for: CD28 Autonomous Signaling Orchestrates IL-22 Expression and IL-22-Regulated Epithelial Barrier Functions in Human T Lymphocytes
Source: Front Immunol. 2020 Oct 14;11:590964. doi: 10.3389/fimmu.2020.590964 (PMC7592429; doi:10.3389/fimmu.2020.590964)
Supplement: Supplementary file 2 [file DataSheet_2.pdf]

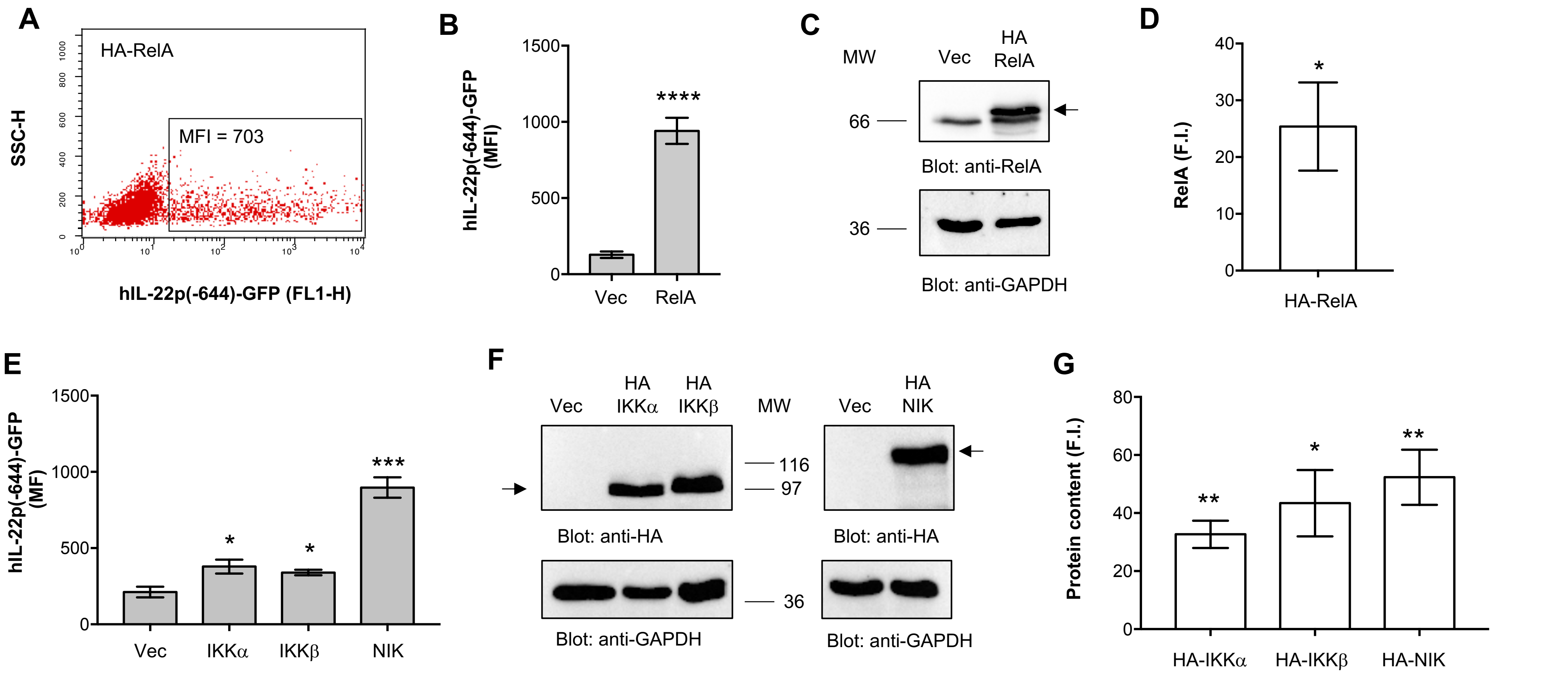

Figure S2. Regulation of IL-22 promoter transactivation by RelA/NF-κB and NF-κB regulating kinases. (**A**) CD28-positive Jurkat cells were transfected with hIL-22p(-644)-GFP construct together with control vector (Vec) or HA-RelA for 24 h and analysed by flow cytometry. A representative dot plot with mean fluorescent intensity (MFI) values calculated within the gated GFP-positive cells (FL-1) of HA-RelA transfected cells is shown. (**B**) Data show the mean MFI ± SEM of four independent experiments. Statistical significance was calculated by Student t test. (**C**) Anti-RelA and anti-GAPDH western blotting of total extracts from Jurkat cells transfected as in (**B**). Arrows indicate the position of HA-RelA. (D) RelA fold inductions (F.I.) over the basal level of cells transfected with Vec were quantified by densitometric analysis and normalized to GAPDH levels. Data express the mean F.I. ± SEM of four independent experiments. Significance was calculated by Student t test. (**E**) MFI of Jurkat cells transfected with hIL-22p-GFP together with control vector (Vec) or HA-IKKα, or HA-IKKβ or HA-NIK. Bars show the mean ± SEM of three independent experiments. Statistical significance was calculated by Student t test. (**F**) Anti-HA and anti-GAPDH western blotting of total extracts from Jurkat cells transfected as in (**E**). Arrows indicate the position of HA-IKKα, HA-IKKβ and HA-NIK. (**G**) Protein fold inductions (F.I.) over the basal level of cells transfected with Vec were quantified by densitometric analysis and normalized to GAPDH levels. Data express the mean F.I. ± SEM of three independent experiments. Significance was calculated by Student t test. The position of molecular weight markers (MW) is indicated. *$p < 0.05$, ***$p < 0.001$, ****$p < 0.0001$.
